# Disposable Paper-Based Biosensors: Optimizing the Electrochemical Properties of Laser-Induced Graphene

**DOI:** 10.1021/acsami.2c06350

**Published:** 2022-06-29

**Authors:** Gourav Bhattacharya, Sam J. Fishlock, Shahzad Hussain, Sudipta Choudhury, Annan Xiang, Baljinder Kandola, Anurag Pritam, Navneet Soin, Susanta Sinha Roy, James A. McLaughlin

**Affiliations:** † School of Engineering, Ulster University, Newtownabbey, Belfast BT37 0QB, Northern Ireland, U.K.; ‡ Department of Physics, School of Natural Sciences, Shiv Nadar University, Gautam Buddha Nagar 201314, Uttar Pradesh, India; § IMRI, University of Bolton, Deane Road, Bolton BL3 5AB, U.K.; ∥ Department of Chemistry, Indian Institute of Technology Kanpur, Kanpur, Uttar Pradesh 208016, India

**Keywords:** porous graphene, laser-induced graphene, ePAD, electrochemical sensing, uric acid

## Abstract

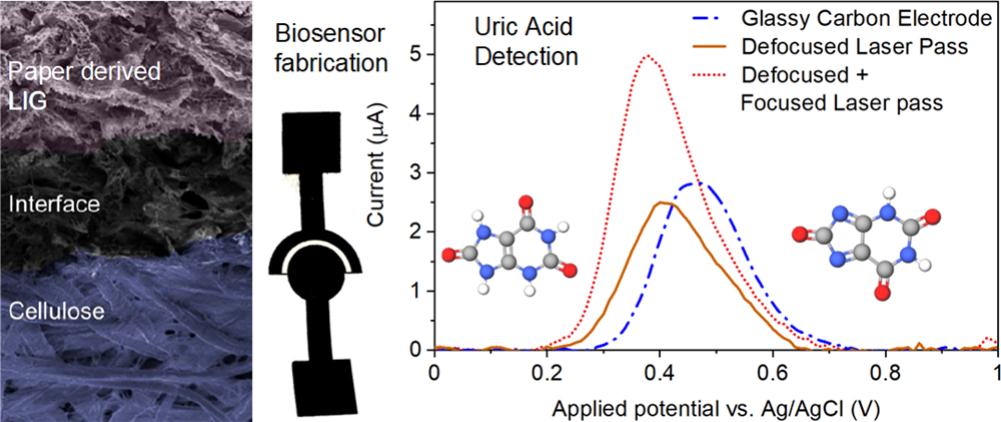

Laser-induced graphene (LIG) on paper substrates is a desirable material for single-use point-of-care sensing with its high-quality electrical properties, low fabrication cost, and ease of disposal. While a prior study has shown how the repeated lasing of substrates enables the synthesis of high-quality porous graphitic films, however, the process–property correlation of lasing process on the surface microstructure and electrochemical behavior, including charge-transfer kinetics, is missing. The current study presents a systematic in-depth study on LIG synthesis to elucidate the complex relationship between the surface microstructure and the resulting electroanalytical properties. The observed improvements were then applied to develop high-quality LIG-based electrochemical biosensors for uric acid detection. We show that the optimal paper LIG produced via a dual pass (defocused followed by focused lasing) produces high-quality graphene in terms of crystallinity, *sp*^2^ content, and electrochemical surface area. The highest quality LIG electrodes achieved a high rate constant *k*_0_ of 1.5 × 10^–2^ cm s^–1^ and a significant reduction in charge-transfer resistance (818 Ω compared with 1320 Ω for a commercial glassy carbon electrode). By employing square wave anodic stripping voltammetry and chronoamperometry on a disposable two-electrode paper LIG-based device, the improved charge-transfer kinetics led to enhanced performance for sensing of uric acid with a sensitivity of 24.35 ± 1.55 μA μM^–1^ and a limit of detection of 41 nM. This study shows how high-quality, sensitive LIG electrodes can be integrated into electrochemical paper analytical devices.

## Introduction

Paper (cellulose and nitrocellulose) is a suitable substrate for a range of electronic applications, including sensors for healthcare and environmental monitoring.^1^ As a material, paper has a long history of utilization as a substrate in passive analytical applications such as pH indicator strips and lateral flow assays—particularly for pregnancy tests^2^—and more recently as rapid COVID-19 Antigen tests.^3^ Recently, paper substrates have been finding increasing use in electronic and electrochemical devices with their inherent advantages of low cost, flexibility, fluid handling, and simple disposal.^4,5^

Paper substrates often fill a niche where a trade-off between cost and performance is required, and as a result, a large amount of inventive research is focused on improving the performance. One important application is for electrochemical paper-based analytical devices (ePADs) in healthcare and environmental sensors.^6^ ePADs operate where a liquid analyte passively flows by the capillary action of the paper to an electrode, which is used to detect the presence and quantity of the analyte. ePADs are well suited for detecting electroactive substances including ions,^7^ dopamine,^8^ and other biomarkers such as uric acid and ascorbic acid^9^ as measurements can be taken simply at the point-of-care and then the device can be disposed of. Uric acid is particularly important as a biomarker for gout—an inflammatory disease—as it forms crystals of monosodium urate, which builds up in joints.

Beyond ePADs, there is increasing research into wider applications for paper-based electronics, which have been comprehensively reviewed by Tai et al.,^11^ who showed that the rapidly growing field of paper-based physical, gas, and humidity sensors demonstrates great promise due to the chemo-physical properties of the underlying paper, including flexibility and water absorption. Further innovations include multifunctional paper-based sensors, which use a single physical sensing mechanism (humidity) to measure multiple useful parameters such as respiration rate, skin moisture sensor, and also use as a physical switch.^12^ Duan et al. also showed a paper-based multifunctional sensor using carbon ink^13^ for humidity and pressure sensing. They showed that the rough, fibrous structure of paper coupled with its hydrophilicity and flexibility endowed the sensors with remarkable sensitivity while simultaneously reducing e-waste since the paper-based sensor can be harmlessly disposed.

There are various methods of producing electronic devices from paper substrates, the most common of which has been screen printing, which has low cost and is scalable. A screen/mask is used to pattern conducting inks such as carbon.^13^ The ability to design and print patterned electrodes using computer-aided design (CAD) software is a significant advantage of inkjet printing over screen printing as prototype designs can be rapidly trialed. The number of inkjet materials is rapidly expanding^14^ though there are some limitations on the viscosity, surface tension of materials that can be jetted, and inkjet clogging continues to be a problem with some materials.

Another promising route for “electrifying” paper devices is to use laser treatment to convert cellulose into graphitic carbon. In 1991, Schumann et al. showed the conversion of polymers such as polyimide into a conductive material through treatment by a UV laser.^15^ The Tour group at Rice University used a CO_2_ laser to convert polyimide^16^ and paper/cellulose^17^ among others into porous 3D graphene films called as laser-induced graphene (LIG). This technique enabled the fabrication of detailed graphene patterns onto paper substrates for applications such as sensors.^10,18^ Furthermore, the surface properties of the LIG may be tuned by various methods including by adding solvents to the LIG surface^19^ to create multifunctional surfaces with superhydrophobic properties. LIG has inherently high adhesion to the paper substrate, hence reducing the chance of delamination and improving resistance to strain, which is crucial for flexible sensors in particular.^20^ Paper-derived LIG may also be combined with other high-throughput techniques such as wax printing^21^ to take advantage of the fluid handling properties of paper such that the liquid analyte may be efficiently delivered and concentrated onto the sensing electrode.

Previous studies describing the mechanism of LIG formation have shown how the quality of graphene can be improved through varying the laser power, focus, and the number of repeat runs and that initial defocused lasing followed by focused lasing has shown to improve the quality of graphene films.^17,22^ The well-ordered crystal structure and thermal decomposition enable a good yield of graphitic carbon from cellulosic precursors,^23^ which suggests that this is an ideal electrode material for ePADS. The conversion of fire-retardant-treated cellulose to graphitic carbon via multiple CO_2_ lasing is photothermally driven, where the heating effect releases volatile gases, converting cellulose sequentially to aliphatic and aromatic char and eventually to graphitic carbon.^24,25^ Currently, however, the mechanistic insight into LIG formation and how that correlates to the ensuing surface-driven electrochemical properties, and the microstructural properties within the bulk of the material, is missing. These inherent physico-electrochemical properties drive the electron transfer rate, the fundamental understanding of which can help to reliably produce electrodes which are easily scalable.

In this work, we systematically vary the lasing treatment conditions of cellulose substrates and measure the material and electrochemical changes. To characterize the material changes, we probe the resulting LIG systems for their microstructure using surface-sensitive (XPS), intermediate (Raman), and bulk/depth characteristic (TGA–FTIR) measurements, which provide an authoritative cross section of the paper-based LIG and the resultant physico-electrochemical properties. We show how multiple lasing steps at different focal lengths reduces the surface oxygen content and increases both the *sp*^2^ carbon content and the electrochemical surface area (ECSA) of the LIG. We further show how the commensurate high-performance electrochemical response can be utilized for disposable paper LIG-based biosensors for uric acid detection using chronoamperometry.

## Materials and Methods

The commercially available Whatman filter paper No. 1 was employed for LIG synthesis via laser treatment. Fire-retardant pretreatment is normally required for cellulosic materials to avoid breakdown into volatile chemicals during laser treatment.^17,25^ To treat the paper substrate, a commercial flame retardant (Firechief, UK) was used. Potassium chloride (KCl), phosphate buffer saline (PBS), potassium hexacyanoferrate(III) (K_4_[Fe (CN)_6_]), potassium hexacyanoferrate(II) trihydrate (K_3_[Fe(CN)_6_ 3H_2_O]), and uric acid of AR grade were purchased from Sigma-Aldrich (UK) and used without any further purification. Ultrapure deionized (DI) water (Millipore Milli-Q system) with an electrical resistivity of 15 MΩ was utilized to produce the aqueous solutions.

### Synthesis of LIG

To prevent the laser irradiation-induced thermal decomposition of paper into volatile compounds, the filter paper was briefly soaked (5 min) in a fire-retardant solution and subsequently dried overnight under ambient conditions. The thus-obtained treated filter paper was irradiated using a 10.6 μm CO_2_ laser cutter (Universal Laser 230 VLS) for which a simple 25 × 25 mm^2^ design was produced using AutoCAD (Autodesk 2017, USA). The subsequent raster scan-based lasing operation was carried out at a pulse separation of 25.4 μm (1000 PPI), 1.5 W power, and a scan speed of 15 cm s^–1^. These conditions were made based on our initial trials and previous study^26^ for which high-quality graphene films were confirmed using Raman analysis. From our results, we found that using relatively higher fluences lead to films which can easily delaminate or become “dust like,” while relatively lower powers will lead to a network of LIG that is not fully developed with some regions of amorphous carbon or even unconverted cellulose that are electrically insulating. Our findings match the parametric studies performed by other groups^21^ who have reported that optimum experimental conditions yield a uniform and dense LIG network, while higher fluences can lead to ablation of the network.

We varied the focal length distance and the number of lasing cycles to examine their effect on the microstructure of LIG and thus its physico-electrochemical properties. For the samples where the initial lasing was carried out at the laser focal length, the as-produced LIG samples were termed 1F. For the defocused lasing condition (where the substrate is 0.8 mm below the focal point), the sample was named 1D, while the LIG samples prepared using consecutive lasing cycles in defocused and focused regimes were labeled 1D1F. Finally, the sample prepared using two consecutive lasing operations at the focal length was termed 2F.

### Characterization

The surface morphology and microstructure of the samples were studied using a Hitachi SU5000 field-emission scanning electron microscope (FESEM). X-ray photoelectron spectroscopy (XPS) was performed using a Kratos Axis Ultra DLD spectrometer with an Al Kα (*h*ν = 1486.6 eV) X-ray source operating at 15 kV and 10 mA (power = 150 W). While the wide-energy survey scans (WESSs) were obtained at a pass energy of 160 eV, the high-resolution core spectra were recorded at a pass energy of 20 eV. A Kratos charge neutralizer system with a filament current of 2.05 A and a charge balance of 3.8 V was used for all the samples. Sample charging on the positions of the measured binding energy (BE) was corrected by setting the *sp*^2^ component of the C 1s spectral envelope to 284.8 eV. The high-resolution spectra were deconvoluted in CasaXPS using Shirley background subtraction and Gaussian–Lorentzian functions. The Raman spectra were collected using a 532 nm laser-based Renishaw inVia Raman spectrometer with 30 s exposure time at 25 mW laser power.

The thermal degradation behavior and the gaseous analysis of paper and 1D1F samples were studied using the hyphenated thermogravimetric–Fourier transform infrared (TG-FTIR) technique. This study was performed using a TA Instruments SDT 2960 Simultaneous DTA/TGA apparatus using platinum pans under a nitrogen flow of 100 mL/min. Before the tests, all the samples were dried at 80 °C for 4 h, and 10 mg of samples was used for each test. The heating rate was maintained at 10 °C/min and the measurements were carried out from ambient temperature up to 900 °C. Evolved gases from the TGA instrument were collected inside a heated gas line (*T* = 200 °C) connected to an FTIR instrument (Thermo Fisher Nicolet iS10). The temperature and time which gave the maximum degradation for each sample were identified using the DTG curves, and the corresponding FTIR spectrum (absorbance vs wavenumber) at that instance was obtained through the Gram–Schmidt plot. The identification of the evolved gaseous compounds was realized using vendor-provided OMNIC FTIR software.

### Electrochemical Measurements

The cyclic voltammetry (CV) and electrochemical impedance spectroscopy (EIS) measurements were performed using a BioLogic potentiostat/galvanostat SP-200 (BioLogic, France). The CV and EIS scans were conducted in a conventional three-electrode cell configuration with an Ag/AgCl reference, a platinum wire counter, and an LIG-modified glassy carbon electrode (GCE) as the working electrode. The CV scans were recorded between −0.5 and 1.0 V with a 0.1 M aqueous KCl electrolyte solution containing a 5 mM Fe(CN)_6_^3–/4–^ redox couple. The EIS spectra were collected at the open-circuit potential (OCP), and an AC sinusoidal perturbation voltage with an rms value of 10 mV was applied while the frequency was varied from 0.01 Hz to 5 MHz. The obtained data were fitted using vendor-provided ZFit software.

### Square Wave Anodic Stripping Voltammetry (SWASV) and Chronoamperometry-Based Uric Acid Detection

SWASV was employed for the detection of uric acid (10 μM in 0.1 M PBS (pH ∼ 7.4)) using a BioLogic potentiostat/galvanostat SP-200 using a deposition potential of −0.5 V, a deposition time of 60 s, a pulse height of 25 mV, a pulse width of 100 ms, and a step height of 10 mV.

To demonstrate the real-time suitability of the paper-derived 1D1F sensor for uric acid detection, chronoamperometry using a two-electrode sensor assembly was employed (the developed sensor with dimensions is shown in Figure S1), and the assembly was pasted onto a polymer substrate. The sensor was designed to ensure that the liquid analyte is suitably concentrated within the sensing region. To achieve this, the outline of the LIG was ablated and removed from the surrounding paper section. This ensured that the only remaining unlased paper was that remaining between the two electrodes, and no liquid flowed into regions outside of the sensing area.

A two-electrode sensor assembly was used to determine the linear range and sensitivity of the LIG sensor to uric acid via chronoamperometry. For these measurements, uric acid (1–1000 μM) solutions in 0.1 M PBS were prepared and 2 μL of the test solution was applied to the sensor’s unexposed area while continually measuring the current for 40 s (at an applied potential of 0.5 V vs OCP). To study the current response as a function of uric acid concentration, after every 60 s, 2 μL of uric acid solution with incremental concentrations was added.

## Results and Discussion

### Morphology Analysis

**Figure 1:**
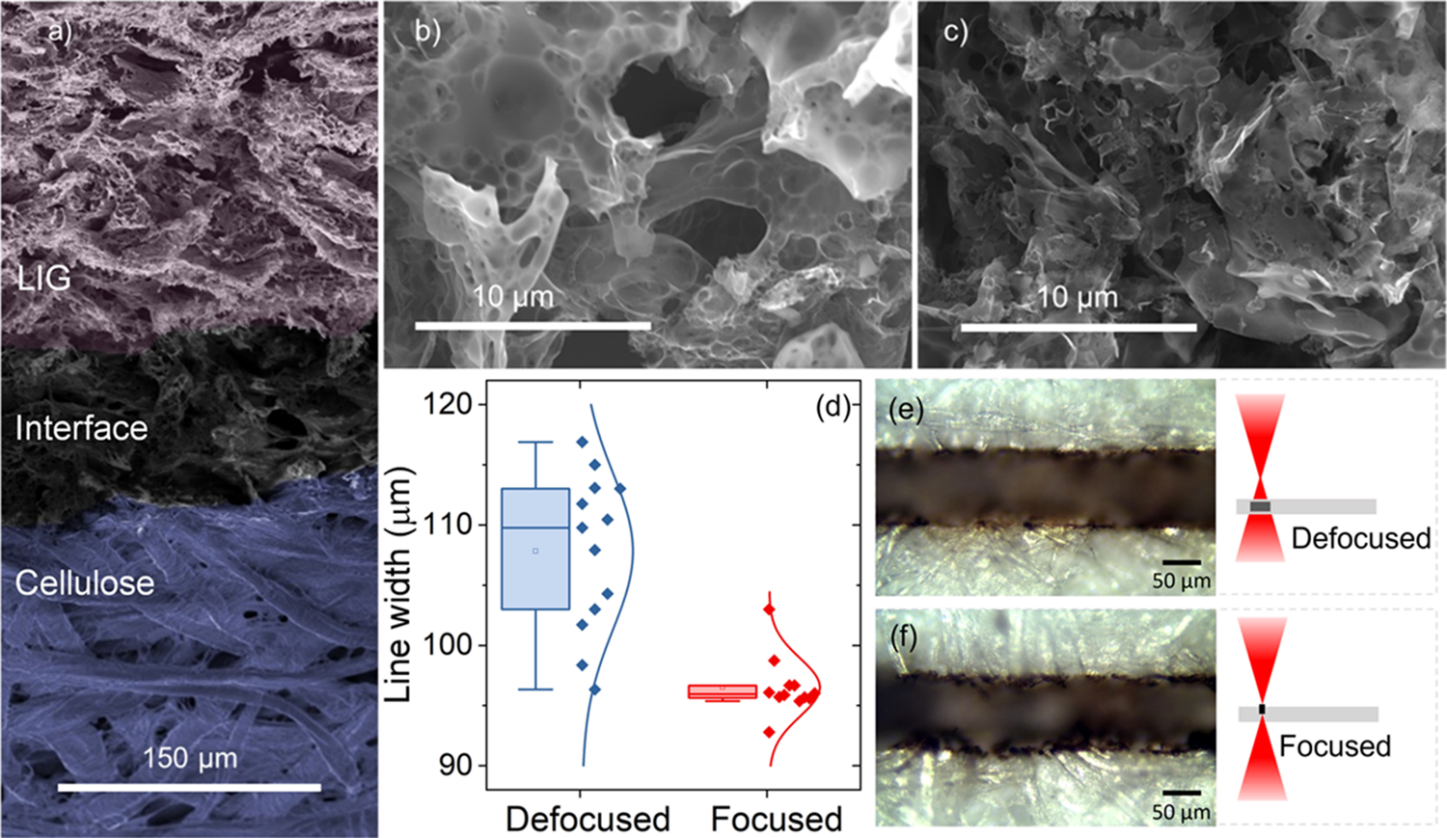
Scanning electron microscopy (SEM) morphological images of the LIG samples showing (a) false color image of the interface between the pristine paper substrate and the obtained 1D1F LIG at a 60° tilt angle, (b) SEM image of 1D paper-derived LIG, (c) SEM image of 1D1F paper-derived LIG, (d) analysis of the ablation width of a cellulose substrate under defocused and focused conditions, and (e, f) optical microscopy images showing the slight increase in the ablation width from a defocused laser pass.

Upon lasing, all four LIG samples (1D, 1F, 1D1F, and 2F) showed a dramatic change in their morphology (Figure 1a), wherein the cellular, fibrous nature of the pristine paper was converted to porous nanostructured carbon with visible flake-like features as seen in all samples, see Figures 1b,c and S2a–c. To understand the differences arising from the focusing conditions, we have measured the relative ablation width for the defocused (107.8 ± 6.5 μm) and focused lasing (96.5 ± 2.4 μm) regimes on the paper substrate (see Figure 1e,f). While the overall amount of energy transmitted to the substrate is the same in both cases, under the defocused condition, the area exposed to the laser is relatively higher, which therefore reduces the laser fluence.^27^ Nevertheless, despite the lower fluence, we still exceeded the critical fluence energy required for the initiation of the carbonization process on paper.

### Raman Spectroscopy

**Figure 2:**
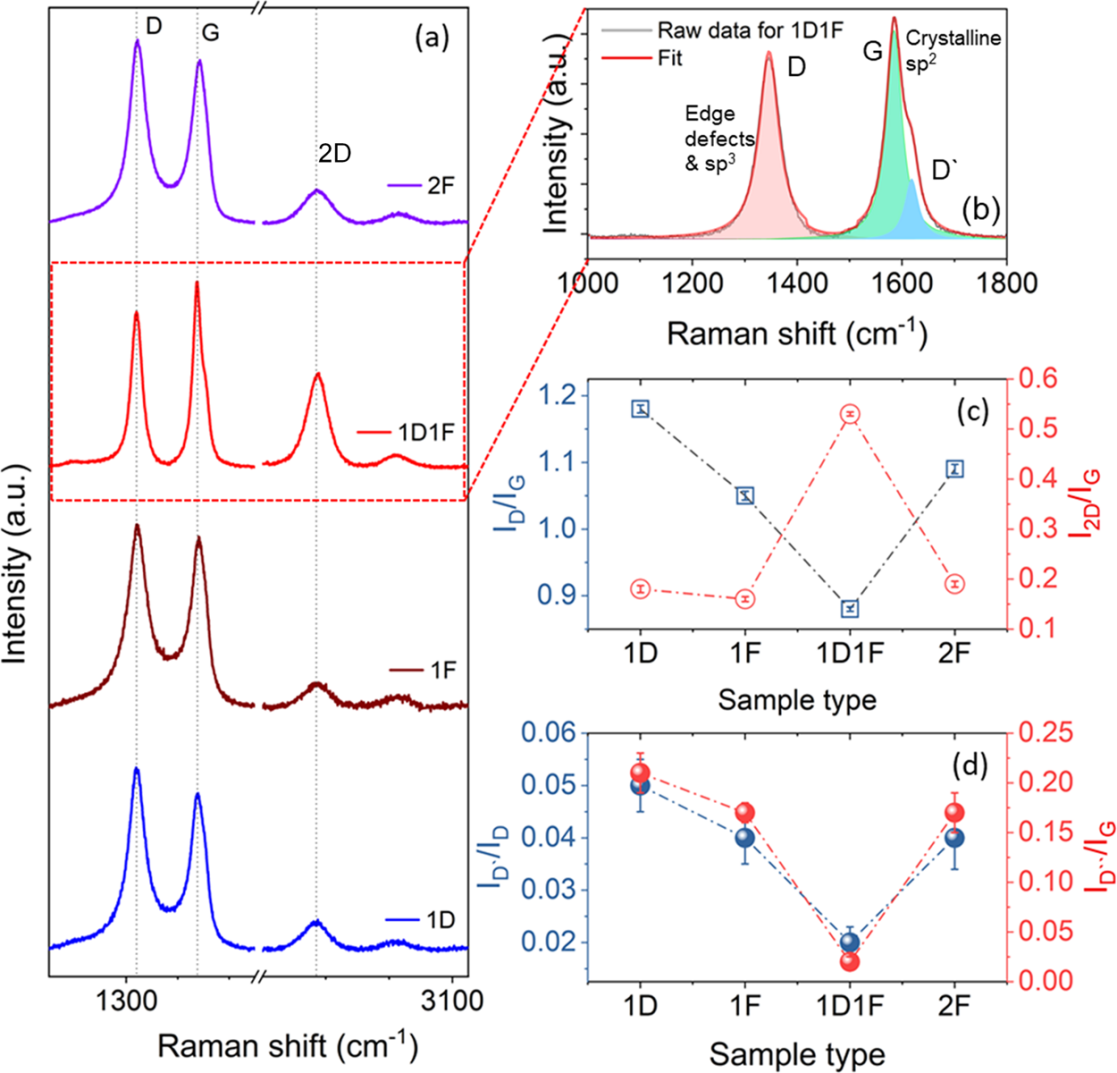
(a) Raman spectra for each of the four samples. (b) Deconvolution of the Raman spectrum of 1D1F into D_sp2_, G, and D′ peaks. (c) Graph showing the *I*_D_/*I*_G_ and *I*_2D_/*I*_G_ ratios for the four LIG samples. (d) Graph showing the Raman spectroscopy ratios: *I*_D*_/*I*_D_ and *I*_D″_/*I*_G_ for the four LIG samples.

The nature and the quality of all paper-based LIGs were studied using Raman spectroscopy and are plotted in Figure 2a. All the samples exhibited three major characteristic graphitic peaks (D, G, and 2D) located at ∼1350, ∼1580, and ∼2700 cm^–1^, respectively, in line with the earlier study.^28^ We have further deconvoluted the first-order Raman spectra for the samples as shown (for the 1D1F sample) in Figures 2b and S2a–c (discussed later). The comparison of quality of the as-produced LIGs was investigated by the *I*_D_/*I*_G_ and *I*_2D_/*I*_G_ ratios and is shown in Figure 2c. The *I*_D_/*I*_G_ (∼1.13) ratio was the maximum in the case of the 1D sample indicating the highly defective nature of the obtained LIG. The disordered nature and structural defects were reduced for other samples and the 1D1F sample had the lowest *I*_D_/*I*_G_ ratio of 0.88 compared with 1.05 and 1.09 for 1F and 2F samples, respectively.

It has been reported earlier that cellulose, which consists of aliphatic carbons, can be decomposed easily upon laser irradiation to a highly defective LIG structure.^17^ It has also been shown that a defocused laser operation has a relatively poor conversion rate of the cellulose into LIG, and thus a highly defective carbon structure is produced after one lasing cycle. However, a two-step laser irradiation process initially photothermally converts the paper into amorphous-rich carbon and in the subsequent exposure that amorphous-rich carbon is transformed into graphene, and therefore, multiple laser irradiation induces a high-quality graphene structure, which was observed for the 1D1F specimen (with the lowest *I*_D_/*I*_G_ ratio).

An opposite trend was observed in the case of the *I*_2D_/*I*_G_ ratio (Figure 2c). The second-order 2D peak, which arises due to the two-phonon resonance, is a fingerprint of the number of layers in graphene-based materials.^29^ A higher and more prominent 2D peak can be ascribed to fewer layers of graphene within each crystallite.^30^ The lower ratio for 1D, 1F, and 2F samples can be ascribed to multilayer structures with less *sp*^2^ graphitization. The maximum *I*_2D_/*I*_G_ ratio of ∼0.53 was measured for 1D1F, confirming a relatively less number of graphene layers compared with other samples and a higher level of graphitization.^31^ The full width at half maximum (FWHM) of D, G, and 2D peaks was also evaluated, wherein a reduction of FWHM for the G peak from 59 to ∼46 cm^–1^ was observed for 1D and 1D1F samples, respectively, which indicates the enhanced *sp*^2^ content in 1D1F (also confirmed via XPS).^32^ The FWHM of the 2D peaks for all the samples is greater than 70 cm^–1^, which implies a 3D structure of randomly stacked graphene layers along the vertical axis (*c*-axis).^16^

The detailed nature and quality of graphene in the laser-irradiated samples were further examined thoroughly by deconvoluting the D and G peaks of the Raman spectra using a 5-peak model described in the literature.^33,34^ The conventional G-peak (centered at ∼1580 cm^–1^) arises due to the *E*_2g_ vibrational symmetry of an ideal graphitic lattice, while the D_sp2_ peak (centered at ∼1350 cm^–1^) arises due to the presence of a disordered graphitic lattice, which arises because of defects in *sp*^2^-hybridized carbon in the hexagonal graphite lattice.^35^ The D′ peak centered around ∼1620 cm^–1^ results from the disorder-induced phonon vibration originating from crystal defects.^36^ The D* peak, centered between 1100 and 1200 cm^–1^, originates from the edge defects in *sp*^2^*–sp*^3^ hybridization for the disordered graphitic lattice.^37^ The D″ peak (∼1500 to 1550 cm^–1^) is related to the amorphous nature of the graphite lattice^38,39^ and given the higher degree of crystallization and *sp*^2^ content, for the 1D1F sample, this peak is absent but is visible for other samples (see Figure S3).

The intensity ratio of D* and D_sp2_ can provide valuable information related to the *sp*^3^ and *sp*^2^ contents in the LIG films.^40^ The ratio was calculated and plotted in Figure 2d, which shows the highest ratio of ∼0.05 for 1D, indicating a higher *sp*^3^ content. The ratio is identical for 1F and 2F samples, indicative of the relatively higher *sp*^3^ carbon content. Correspondingly, a 2.5-fold reduction in the ratio was observed for the 1D1F sample suggesting a much lower *sp*^3^ content and higher electronic conductivity (confirmed via XPS and electrochemical studies).

The degree of crystallization was further evaluated from the *I*_D″_/*I*_G_ ratio (Figure 2d).^40^ The ratio is maximum (∼0.21) for the 1D sample, which indicates the partial conversion of cellulose into LIG and the presence of relatively higher levels of amorphous carbon. There is a remarkable reduction of the ratio in the case of 1D1F, and the *I*_D″_/*I*_G_ ratio was evaluated to be 0.02, which implies a higher graphitic nature with a much less amorphous nature. The ratio again remains identical for 1F and 2F samples (0.17). In 2F, though two-step lasing was carried out, it is believed that the initial irradiation (1F) produces less amorphous carbon, and the second lasing does not have any significant role in subsequent graphitization and the alteration in the structural properties. The identical values of the *I*_D″_/*I*_G_ ratio and the results obtained by all the electrochemical studies confirmed this observation.

### X-Rray Photoelectron Spectroscopy

**Figure 3:**
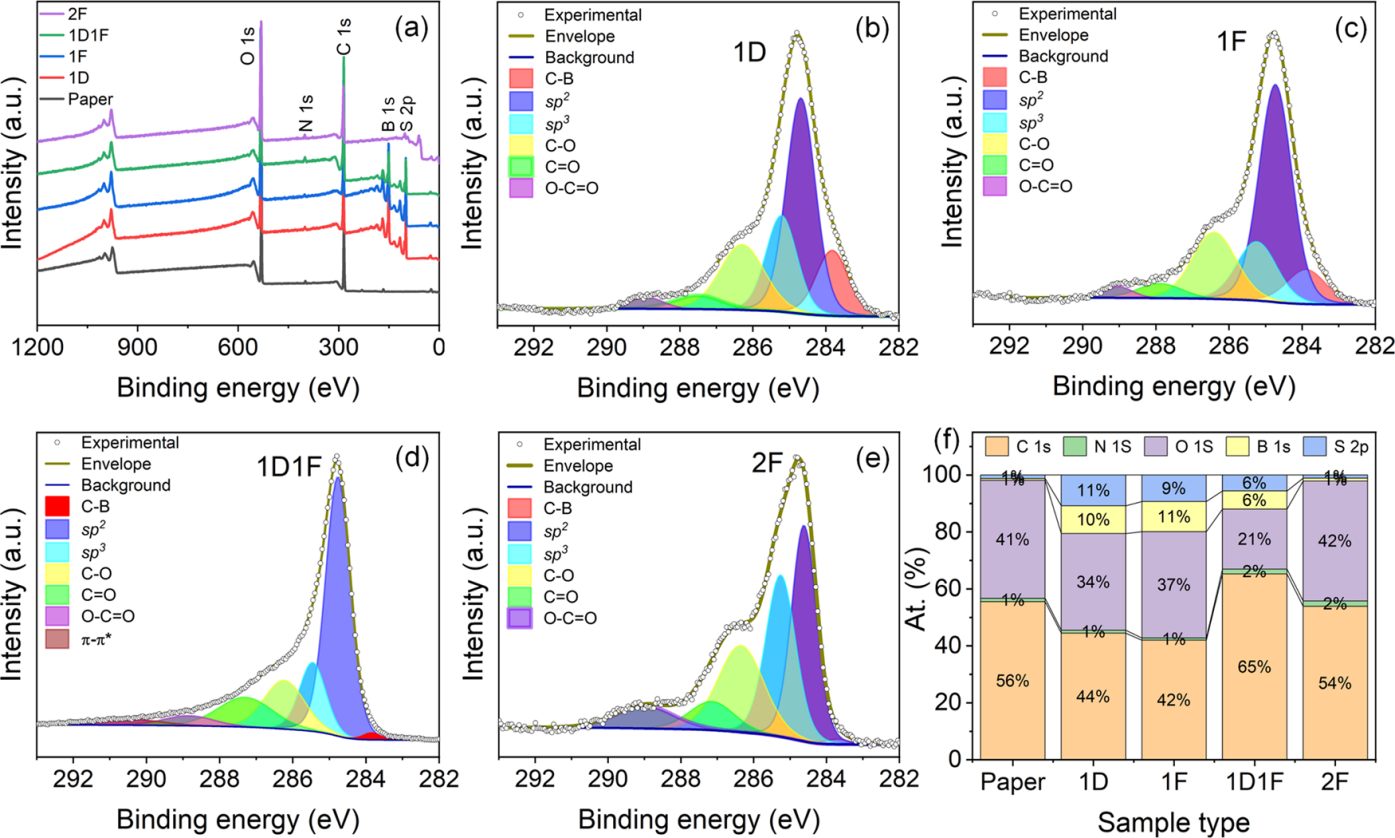
(a) WESS comparison, high-resolution C 1s XPS spectra for (b) 1D, (c) 1F, (d) 1D1F, and (e) 2F samples and (f) atomic % of elements in samples.

The elemental composition changes and the quality of the LIG samples, as a function of laser irradiation, were investigated using XPS analysis. The WESS (Figure 3a) reveals the presence of the elements C, O, N, B, and S for all 1D, 1F, 1D1F, and 2F samples. The detection of boron is attributed to the fire-retardant spray in which the paper substrates were soaked. The carbon content was minimum for the 1D sample (∼41.95%) and maximum for 1D1F (65.24%). The atomic ratios of C and O (C/O) too were evaluated and the 1D1F sample exhibited the highest value (3.09), indicating the lowest oxygen content and consequently a higher electronic conductivity. Interestingly, we observed a higher boron content in 1D and 1F samples compared to the pristine fire-retardant soaked paper. We speculate that the surface enrichment by boron occurs during the initial lasing operation, whereas for the fire-retardant soaked paper, boron is largely absorbed in the bulk of cellulose. This is a common phenomenon that has been reported previously in the literature.^26^ For the 2F sample, the relatively higher photothermal energy (owing to higher fluence) may lead to desorption and removal of boron, which was measured at a relatively lower level (1 atom %) vs 6 atom % for the 1D1F samples.

The high-resolution C 1s spectra for 1D, 1F, 1D1F, and 2F are plotted in Figure 3b–e. All the spectra exhibited an asymmetric nature, indicating the coexistence of other chemical groups on the respective surfaces. The core-level spectra for the samples were deconvoluted using six different regions; furthermore, an extra π–π* region was necessary for the 1D1F sample. The major peak at 284.7 ± 0.1 eV represents the C=C *sp*^2^ bonding, while the small shoulder at 283.8 ± 0.1 eV corresponds to C–B bonding, respectively.^41^ The peaks centered at 285.3 ± 0.2, 286.4 ± 0.15, 287.4 ± 0.2, and 288.9 ± 0.1 eV correspond to C–C, *sp*^3^; C–O; C=O; and O–C=O groups, respectively.^26^ Furthermore, a peak centered at 290.5 eV detected for 1D1F corresponds to the π–π* feature.^42^ The existence of the π–π* shakeup feature confirms that further carbonization and aromatization occur for 1D1F, further improving the electrical conductance compared to the other samples.^43^ The evolution of different chemical groups indicative of changes in the surface chemistry of the synthesized materials is presented in Table S1 (and presented in Figure 3f). The *sp*^2^ fraction, C=C, is maximum (50.15%) for the 1D1F sample, which agrees with our finding in Raman measurements. Furthermore, 1D1F exhibited the least *-sp*^3^ and C–O bonding. The high *-sp*^3^ content in the 2F sample may be attributed to the fact that during the two consecutive laser irradiation steps at the focal height, the LIG may be physically damaged, which induces more *sp*^3^-type defects.

### TG-FTIR

The lasing-induced changes in the chemical structure of selected samples were further investigated using hyphenated thermogravimetric analysis coupled with Fourier transform infrared spectroscopy (TGA–FTIR). For the sake of brevity, we only analyzed the paper and the 1D1F sample (which showed the highest electrochemical response, discussed in the next section). For both the samples, a similar degradation curve of single-step weight loss was observed in the range of 200–400 °C (Figure S4a). The corresponding differential thermogram (DTG) confirmed a maximum degradation temperature of 249 °C for paper, which was lower than that (255 °C) of the 1D1F sample (Figure S4b). Thus, the thermal stability of the paper was enhanced upon the lasing treatment, which was also reflected in the slightly enhanced char residue for the 1D1F sample (31.2% vs 29.9% for paper at 900 °C).

**Figure 4:**
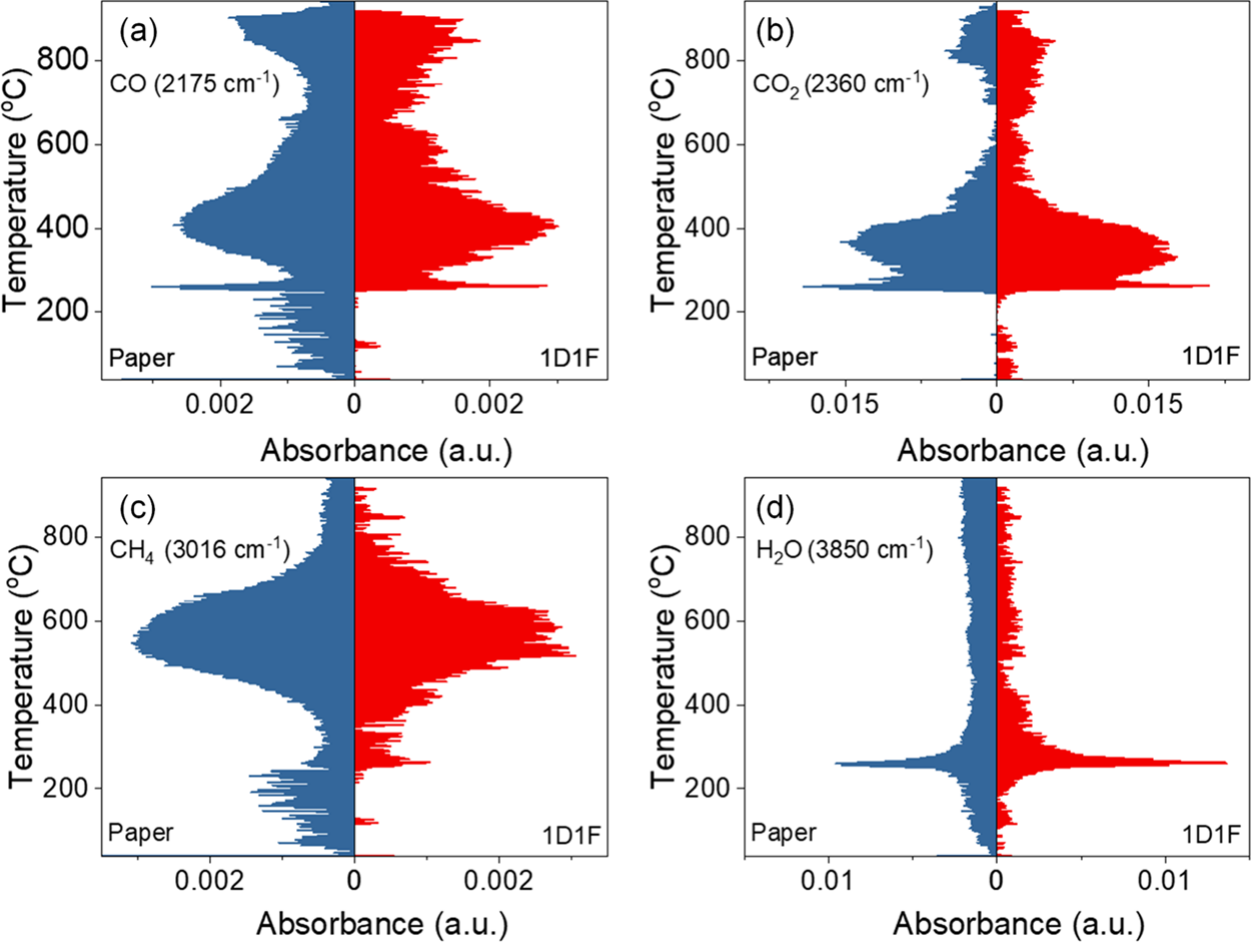
Release profiles of (a) CO, (b) CO_2_, (c) CH_4_, and (d) H_2_O as a function of temperature for both the FR-treated Whatman paper sample and the 1D1F sample.

The coupled TGA–FTIR analysis showed that both the samples exhibited a predominant release of CO_2_, CO, CH_4_ or other hydrocarbons, and H_2_O as shown in Figure 4a–d. Generally, most volatiles were detected in the 200–400 °C temperature range, consistent with the main area of thermal decomposition, which matches well with the reported decomposition of cellulose.^44^ The comparative concentrations of the identified substances as a function of temperature (Gram–Schmidt plots) for the paper and 1D1F samples are plotted in Figure S4b. The evolution of CO_2_ for both samples continued over a long period between ∼250 and 900 °C with peaks at 270, 360, and ∼820 °C for paper and 850 °C for 1D1F, respectively. The release of CO showed a similar trend to that of CO_2_ albeit at a lower concentration. It should be mentioned that the higher release temperature for both CO and CO_2_ for the 1D1F sample can be correlated to the stable and highly crystalline LIG structure. Further calculation of the total carbon and oxygen ratios for the paper and 1D1F species revealed values of (C:O)_1D1F_:(C:O)_paper_ to be ∼1.21. The higher C:O ratio in the case of the 1D1F sample follows the XPS results. It should be noted that as the TGA–FTIR analysis was carried out without isolating LIG from the paper surface, a significant contribution from the underlying paper is expected, which skews the observed values. The evolution of H_2_O was also observed where a higher and sustained release was observed for the paper sample compared to the 1D1F sample. Similarly, the evolution of NH_3_ started at a much lower temperature for paper (∼160 °C) compared to ∼180 °C for the 1D1F sample with the corresponding peak release temperatures of 240 and ∼260 °C, respectively, as shown in Figure S4c. As discussed earlier, both the Raman and XPS measurements confirmed that the 1D1F sample possesses higher crystallinity and is, therefore, more thermally stable requiring a higher temperature for the corresponding release of the gaseous entities.

## Electrochemical Analysis

### Cyclic Voltammetry

**Figure 5:**
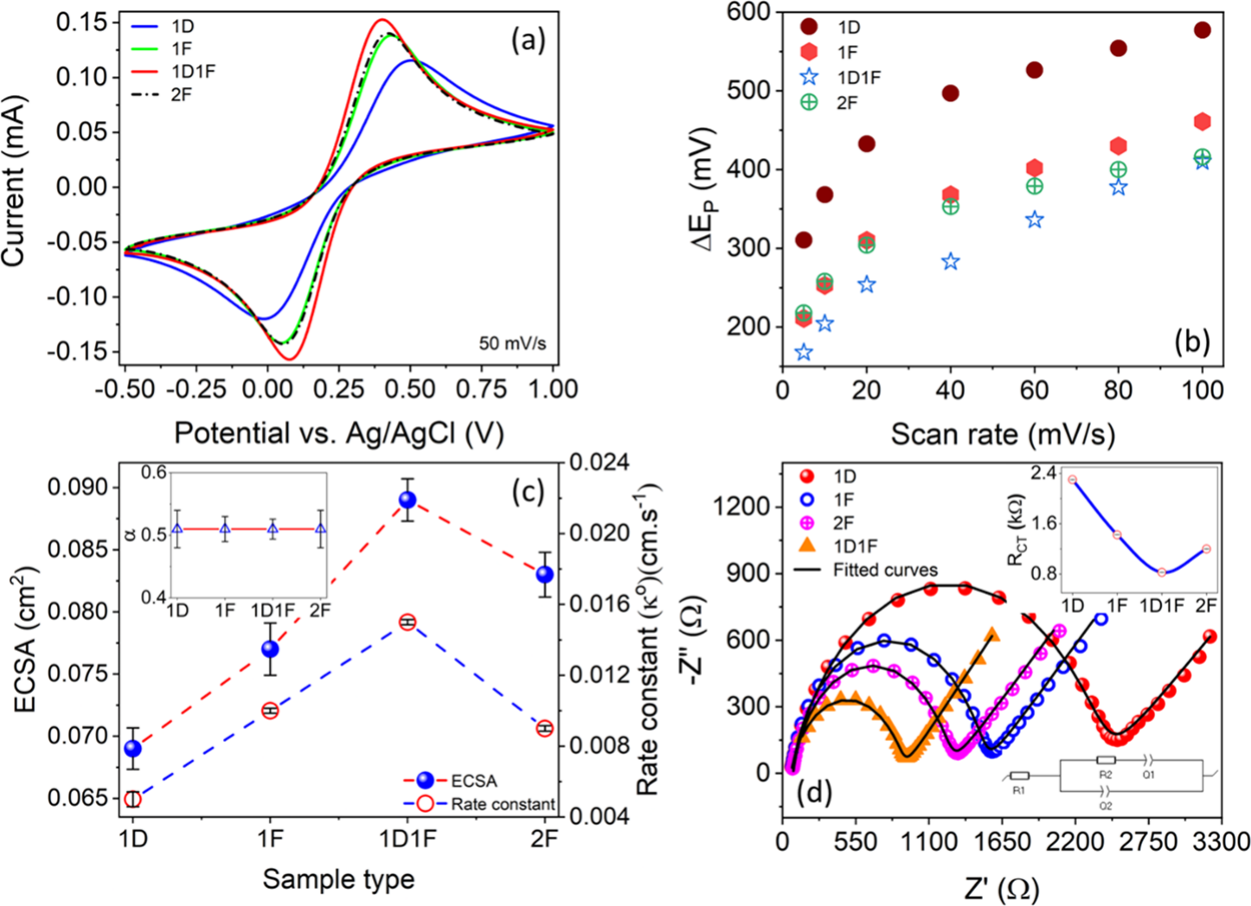
Electrochemical properties of the LIG samples. (a) CV curves for the various LIG samples at a 50 mVs^–1^ scan rate. (b) Separation potential for each sample at varying scan rates. (c) ECSA and rate constant *k*_0_ for each sample with α in the inset. (d) EIS spectra for all samples in 0.1 M aqueous KCl electrolyte solution containing 5 mM Fe (CN)_6_^3–/4–^ with the charge-transfer resistance *R*_CT_ for each sample (inset) along with the circuit diagram for the fitted model.

The cyclic voltammograms of 1D-, 1F-, 1D1F-, and 2F-modified GCE electrodes in the potential range of −0.5 to 1.0 V at a scan rate of 50 mVs^–1^ are presented in Figure 5a. A clear set of cathodic and anodic peaks corresponding to the Fe(CN)_6_^3–/4–^ redox couple can be seen for all the samples. The current density is the minimum for the 1D sample, whereas the maximum current response is observed for the 1D1F sample. The current densities of both the 1F and 2F samples are almost identical and lie in between those of the 1D and 1D1F samples. This trend remains unaltered both at low (10 mVs^–1^) and high (100 mVs^–1^) scan rates (Figure S5), indicating the stability of the measurements and indeed the samples. From the increased peak current, it is evident that the electron transfer of Fe(CN)_6_^3–/4–^ is fastest for 1D1F, indicating the highest electrical conductivity, due to the presence of the highest amount of *sp*^2^-bonded carbon.^45^ The slow electron transfer of the 1D-modified electrode and its relatively poor electrical conductivity are commensurate with the least conversion of paper into LIG and is further confirmed by the presence of most oxygen-containing groups, as analyzed by XPS. The scan rates were further varied from 5 to 100 mVs^–1^ and the CV spectra were subsequently recorded for all the samples.

The separation potential (Δ*E*_p_) which is the difference between the anodic peak potential (*E*_an_) and the cathodic peak potential (*E*_ca_) was calculated for all the samples and plotted against the scan rate (Figure 5b). For all the samples Δ*E*_p_ was the minimum at a low scan rate and increased with higher scan rates. At a fixed scan rate, Δ*E*_p_ was the least for the 1D1F sample and maximum for the 1D-modified electrodes, signifying better reversibility of the 1D1F sample. At the lowest scan rate of 5 mVs^–1^, Δ*E*_p_ values of 310, 211, 218, and 167 mV were measured for 1D, 1F, 2F, and 1D1F-modified electrodes, respectively. These values indicate the quasi-reversible electrochemical behavior for all the electrodes and the smallest separation of 1D1F confirms it as a promising candidate for electrochemical sensors and storage devices.^46,47^

The electrochemical behaviors of all the electrodes as a function of scan rate were further explored using the Randles–Sevcik equation (eq 1):
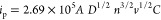
1where *i*_p_ is the peak current, *A* is the ECSA, *D* is the diffusion coefficient, *n* is the number of electrons participating in the charge-transfer process, *v* is the scan rate, and *C* is the concentration of the supporting electrolyte.

The anodic and cathodic peak currents (*I*_pan_ and *I*_pca_, respectively) were then plotted against the square root of scan rate (S6a–e). The curves were fitted and the excellent linear increment for both the anodic and cathodic peak currents was established. This linearity suggests that the redox reaction is controlled by semi-infinite linear diffusion with rapid electron transfer.^48^ Furthermore, eq 1 was used to determine the ECSA from the slope of the *i*_p_ versus *v*^1/2^ plot using the following parameters: *D* (7.17 × 10^–6^ cm^2^ s^–1^ in 0.1 M KCl),^49^*n* (1 for Fe(CN)_6_^3–/4–^ redox couple), and *C* (0.1 M cm^–3^), which is shown in Figure 5c. Following earlier trends, the 1D1F-modified electrode exhibited the maximum surface area of 0.090 ± 0.002 cm^2^ whereas the surface area is least for the 1D-modified electrode (0.070 ± 0.005 cm^2^). The surface areas of 1F and 2F modified electrodes lay in between. Unlike the physical geometric area (0.03 cm^2^), ECSA is the actual area of the electrode material accessible to the electrolyte for charge transfer and/or storage. The increase in the activity ratio (ratio between the ECSA and the geometric area of the electrode) can be correlated with the 3D porous fibrous and flake-like network structure of the LIG (confirmed from SEM), which provides additional accessible active sites for the electrolyte.^21^ Invariably, a higher ECSA is desirable for high-quality electrodes for electrochemical sensing.^50^ The increased ECSA and activity ratio for 1D1F electrodes thus again prove its suitability as a potent electrode material.

To study the in-depth charge-transfer kinetic mechanism, the heterogeneous rate constant (*k*_0_) was further evaluated for all the samples using the Nicholson method^51^ and its extension.^52^ In this method, the Nicolson dimensionless number (ψ) can be defined as shown in eq 2:
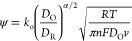
2where *D*_O_ and *D*_R_ are the diffusion coefficients of the oxidized and reduced species, respectively, *R* is the universal gas constant, *T* is the temperature in Kelvin, and *F* is the Faraday constant. The value of the transfer coefficient α can be calculated using the Laviron equation:^53^
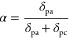
3where δ_pa_ and δ_pc_ are the slopes that were calculated from the anodic and cathodic peak currents versus the logarithm of the scan rate plots, respectively (Figure S7). The values were calculated and the variation of α for all the LIG samples was plotted in the inset of Figure 5c. The average value for all the LIG electrodes is found to be ∼0.5, which signifies symmetrical redox kinetics. If we consider *D*_o_ ≅*D*_R_, then eq 2 can be rewritten as:
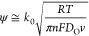
4

Separately, the parameter ψ can also be calculated semi-empirically with the separation potential (Δ*E*_p_):
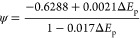
5

Utilizing eqs 4 and 5, the value of *k*_0_ was determined from the slope of ψ versus *v*^–1/2^ (Figure S8). The variation of *k*_0_ for all the LIG samples is plotted in Figure 5c. The rate constant was minimum in the case of the 1D electrode (5 × 10^–3^ cm s^–1^), signifying a slow charge-transfer mechanism owing to its lower electrical conductivity due to the relatively higher oxygen content (confirmed using XPS). There is a twofold increment in rate constants for 1F and 2F electrodes (∼1 × 10^–2^ cm s^–1^), which suggests a faster charge-transfer mechanism. The rate constant was fastest for the 1D1F electrode with *k*_0_ = 1.5 × 10^–2^ cm s^–1^, which indicates the fastest charge-transfer mechanism across the electrode–electrolyte interface. This high value demonstrates that the electrode shows promise as a potentiometric sensor and an energy storage device. The values of *k*_0_ for a similar class of electrodes obtained from the literature are shown in Table 1. The 1D1F electrode exhibited a much improved rate constant, denoting a swift charge-transfer pathway. The reduction in the oxygen content and a much higher -*sp*^2^ carbon content, the least *I*_D_/*I*_G_ ratio, and the reduction in FWHM of Raman spectra in the 1D1F electrode can all be correlated with the highest ECSA and *k*_0_.

**Table 1:** Summary of the Rate Constant *k*_0_ with Comparable Recent Studies

material	*k*_0_ (cm s^–1^)	reference
gold nanoparticle-deposited carbon fiber paper electrode	1.22 × 10^–2^	(54)
chromatography paper-derived LIG	6.85 × 10^–4^	(21)
office paper-derived LIG	4.08 × 10^–4^	
glassy carbon	8.8 × 10^–3^	(55)
carbon paper–glucose oxidase-mediated polypyrrole	2.4 × 10^–4^	(56)
filter paper-derived LIG (1D1F)	1.5 × 10^–2^	present work

### Electrochemical Impedance Spectroscopy

Electrochemical impedance spectroscopy (EIS) was employed to investigate the changes in the ionic conductivity during laser irradiation and to probe the nature of charge-transfer kinetics.^57^ The real part of the impedance was plotted against the imaginary component (Nyquist plot) for all the laser-irradiated samples, which is shown in Figure 5d. The representative curves show a set of semi-circular and linear regions reflecting the charge-transfer^58^ and mass transfer phenomena.^59^ The diameter of the semicircle qualitatively indicates the magnitude of the charge-transfer resistance.^60^ From the graph, it is evident that the charge-transfer resistance is the lowest for the 1D1F sample, which confirms that the ion migration is fastest and the electrical conductivity is maximum, which agrees with our other results.

The EIS data were fitted using a model equivalent circuit represented in the inset of Figure 5d. A modified Randle’s circuit was used to fit the EIS spectra.^61^ The circuit consists of a series resistance (*R*_s_) which is in series with a parallel circuit which is constituted by a resistance (*R*_CT_) and two constant phase elements (*Q*_1_ and *Q*_2_).^62^ The first resistance (*R*_s_) can be considered as the total resistance arising due to contact resistance, material resistance, and the electrolyte.^63^ All the fitting parameters for the LIG samples are tabulated in Table 2. The series resistance, *R*_s_, for all the electrodes was observed to be ∼65 Ω. The variation in charge-transfer resistance obtained from the impedance fitting was further plotted in the inset of Figure 5d. The charge-transfer resistances of 2.29, 1.43, 0.83, and 1.20 kΩ were obtained for the 1D, 1F, 1D1F, and 2F electrodes, respectively. The EIS spectra of the bare GCE electrode are also fitted and an *R*_CT_ value of 1.32 kΩ was acquired. A higher activity ratio, a faster rate constant, a higher *sp*^2^ content, and fewer defects of the 1D1F electrode makes the electron transfer easier, and thus a lower charge-transfer resistance and a higher electrical conductivity are measured. The value is even less than that of a commercially available GCE electrode, which makes the 1D1F electrode a highly attractive candidate for impedimetric sensors.

**Table 2:** Summary of the Fitted Parameters from EIS for All Samples Including a Bare GCE for Comparison

sample	*R*_s_ (Ω)	*R*_CT_ (Ω)	*Q*_1_ (μmho)	*Q*_2_ (mmho)	*Χ*^2^
1D	65	2299	3.5	2.83	0.056
			*n* = 0.81	*n* = 0.39	
1F	65	1425	2.1	3.04	0.014
			*n* = 0.88	*n* = 0.43	
1D1F	65	828	3.9	3.99	0.001
			*n* = 0.86	*n* = 0.47	
2F	65	1199	3.9	3.45	0.065
			*n* = 0.82	*n* = 0.45	
GCE	65	1320	1.88	5.3	0.087
			*n* = 0.90	*n* = 0.53	

### Detection of Uric Acid

**Figure 6:**
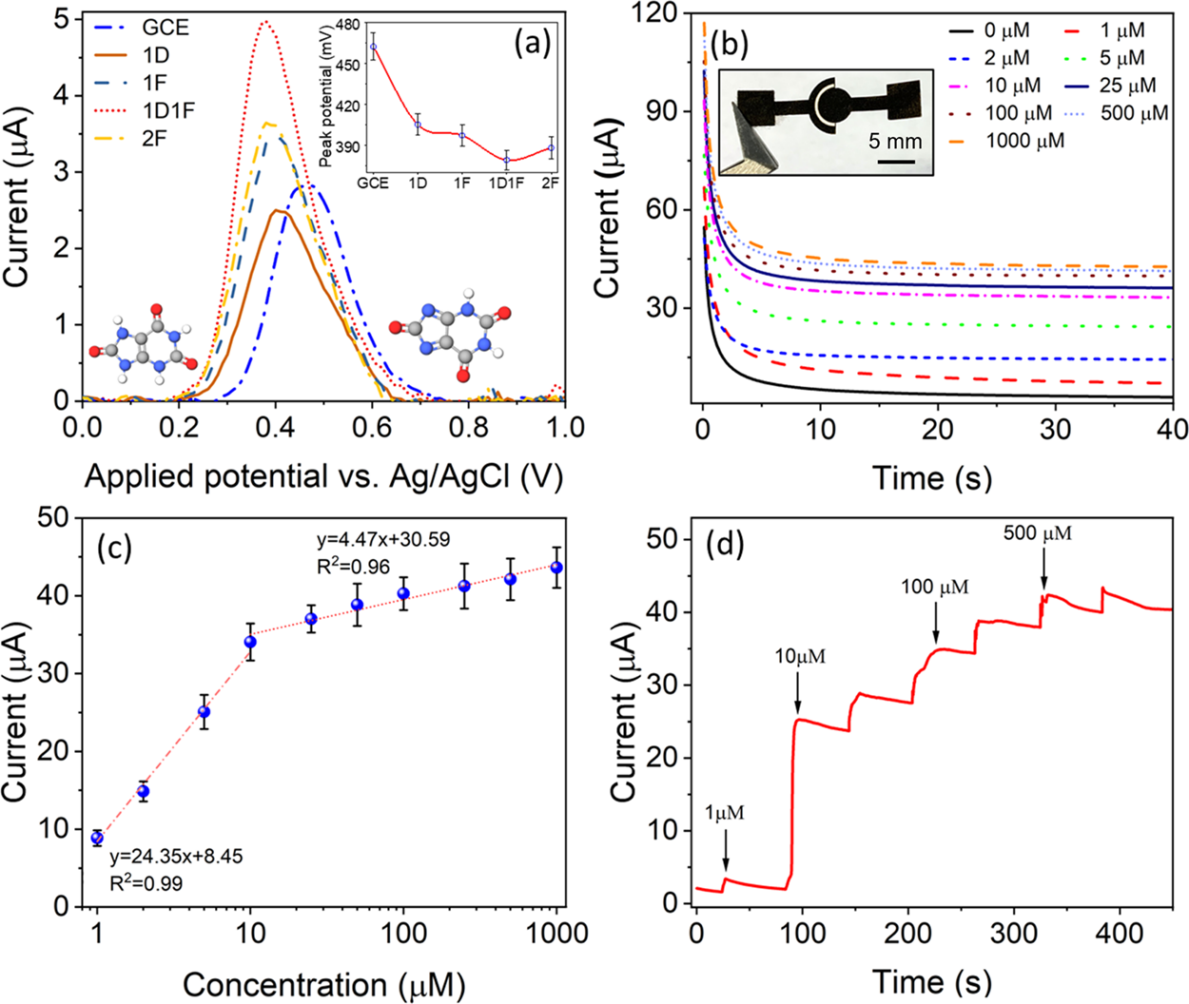
Sensor characterization for the detection of uric acid. (a) SWASV response of LIG-modified GCE to 10 μM uric acid in 0.1 M PBS buffer with the peak potential for each sample shown in the inset. (b) Chronoamperometric measurements of uric acid by a two-electrode 1D1F sample (digital photograph in the inset). (c) Calibration curve of the current–concentration curve, with high and low-concentration regions fitted with linear curves. (d) Dynamic response of the two-electrode 1D1F sensor with incremental increases in uric acid-spiked 0.1 M PBS solution.

Uric acid is an important biomarker for a range of diseases, particularly gout,^64^ and a rapid, low-cost sensor for uric acid detection may help to improve the in-patient resources for assessment and treatment of patients.^65^ Here, we perform SWASV of electrochemical oxidation of uric acid at each electrode as a demonstration of the relative improvement of sensing performance that can be achieved using multiple lasing. The oxidation of 10 μM uric acid in 0.1 M PBS buffer for the electrodes is shown in Figure 5a. The baseline bare GCE electrode exhibited a peak current of ∼2.80 ± 0.10 μA at ∼462 ± 8 mV (vs Ag/AgCl) (*n* = 5). In comparison, the oxidation potential of uric acid for all the LIG-modified electrodes shifted toward a lower potential. This overpotential can be defined as the potential difference between the experimental redox potential and the thermodynamic potential, which is dependent on the analyte concentration and temperature for a specific electrochemical reaction.^66^ Therefore, in the case of bare GCE and all the LIG-modified electrodes, the thermodynamic potential remains identical, while the redox potential can be referred to as the overpotential. The variation in overpotential for GCE and LIG-modified electrodes is plotted in the inset of Figure 6a. The overpotentials for 1D-, 1F-, 1D1F-, and 2F-modified electrodes are measured to be 405 ± 8, 397 ± 8, 379 ± 7, and 388 ± 8 mV, respectively. The overpotential of the 1D1F-modified electrode decreased with a shift of ∼83 mV compared to the bare GCE electrode. As for any electrochemical sensing platform, a lower overpotential is the prime demand, and the 1D1F electrode can be used as a high-quality, stable sensor for the electrochemical detection of not only uric acid but a range of other analytes. The current response of all the electrodes toward the oxidation of uric acid was also monitored. The peak current for the 1D-modified electrode was reduced to ∼2.40 ± 0.10 μA. The presence of abundant oxygen and the partial conversion of paper into LIG can be correlated with its poor conductivity. The peak current for all 1F, 2F, and 1D1F electrodes was higher compared to the bare GCE electrode. For the 1D1F electrode, there is almost a twofold increment in the peak current (4.9 ± 0.1 μA), again confirming its superior electronic conductivity, which is discussed throughout this study. The smallest overpotential and the highest current response of the 1D1F electrode make it an attractive candidate for a low-cost, flexible sensing platform. Related techniques such as differential pulse voltammetry^9^ may also be used to selectively detect uric acid within mixtures of other electroactive species due to different redox potentials of the analytes.

### Chronoamperometry

To show the performance of paper-based 1D1F in a prototype device, the two-electrode ePAD, as shown in the inset of Figure 6b, was employed using chronoamperometry for uric acid detection. To probe the diffusion-controlled current only, and to satisfy the Cottrell equation, the potential was kept at 0.5 V. The current response to different concentrations of uric acid (1–1000 μM) as a function of time was recorded for 40 s and is shown in Figure 6b. A constant increment in current was observed with increasing concentrations, and a stabilized current response was achieved after 30 s, which was subsequently used as the calibration time for the sensor. The blank or baseline current of 3.8 ± 0.3 μA was recorded for the PBS solution without any uric acid. The highest current of 43.6 ± 2.6 μA was obtained for the 1000 μM solution.

To determine the sensitivity and limit of detection (LOD) of the sensor, the stabilized current (current after 30 s) was plotted against the uric acid concentration (Figure 6c). The graph can be divided into two separate regions (low and high concentration regions). These regions can be fitted linearly to determine the sensitivity of the sensor. The low-concentration regime (0–10 μM) exhibited a sensitivity of 24.35 ± 1.55 μA μM^–1^ with an LOD of 41 nM. The second regime exhibited a lower sensitivity of 4.47 ± 0.41 μA μM^–1^. The response time, which we measured as the delay period between the rise of the analyte concentration and the upsurge of the current response (taken at 90% of the whole variation), was found to be less than 7 s for all the uric acid concentrations. To evaluate the dynamic response and to perform the continuous monitoring of uric acid, solutions of incremental concentrations were added and are shown in Figure 6d. From the graph, it was found that initially the current increased steadily with the increasing concentration and finally saturated around 1000 μM, highlighting the wide-range use of the LIG-based sensors.

The comparative performance of the sensor regarding the sensitivity, linear range, and, LOD is shown in Table 3. The favorable performance, cost-effective and rapid fabrication, and lack of harmful chemicals in synthesis make the paper-based LIG a promising platform for future ePADS. The results will particularly help future ePADS that employ LIG as the ability to derive a high-quality sensing electrode, directly from the paper substrate, is highly attractive for low-cost, disposable devices. The LIG synthesis is versatile and can be designed using CAD software, and the same CO_2_ laser can also be used to pattern and cut other features of the device.

**Table 3:** Comparative Table of Sensing Performances of Different Uric Acid Sensors in Terms of Detection Range, Sensitivity, and LOD

matrix	detection range	sensitivity	LOD	reference
uric acid/gold-reduced graphene oxide/indium tin oxide	50–800 μM	86.62 ± 0.19 μA mM^–1^	7.32 ± 0.21 μM	(67)
uric acid/graphene oxide (GO)-5-amino-1,3,4-thiadiazole-2-thiol (ATT)/screen-printed electrode	0.1–10 mM	7.857 μA mM^–1^		(68)
uric acid/screen-printed electrode/paper-based analytical device—reduced graphene oxide/gold	0.20–6.0 mM		180 μM	(69)
uric acid/pencil-marked paper-based analytical device	0.05–1 mM	0.3 mA M^–1^	8 μM	(70)
uric acid/paper-derived LIG	1–1000 μM	24.35b ± 1.55 μA μM^–1^	41 nM	this work

## Conclusions

In conclusion, we demonstrated an in-depth material understanding of the paper-derived LIG and the detailed effects of multiple lasing passes in different focusing regimes. We characterized the obtained samples and showed through a range of microstructural analysis and electroanalytical techniques that an optimal synthesis condition of a defocused pass followed by a focused laser pass (termed as 1D1F) yields the highest quality porous graphene (with the lowest oxygen content, highest *sp*^2^ levels, and largest ECSA). The 1D1F samples benefited from the two-stage synthesis with the highest current density and improved electron transfer properties in electrochemical analysis. The derived process–property relationship for the optimal samples was further validated by performing a three-electrode electrochemical SWASV sensing experiment for uric acid, which exhibited reduced overpotential and increased peak current signals. We further utilized the 1D1F LIG electrode into a disposable paper-based, two-electrode chronoamperometric biosensor to demonstrate its application as a high-quality material for ePADS, with a linear response and LOD of 41 nM. The low-cost, flexible material of paper-derived LIG, which may be easily disposed of, exhibiting excellent performance, is an attractive platform for future ePADS.

## Supporting Information Available

Schematic of the two-electrode sensor, SEM images of LIG and pristine paper, table for deconvoluted high-resolution XPS spectra, deconvoluted Raman spectra, pyrolysis curves of pristine paper and 1D1F samples, TGA–FTIR curves for pristine paper and 1D1F sample, CV scans at different scan rates, variation of anodic and cathodic peak currents as a function of square root of inverse of scan rate, variation of anodic and cathodic peak currents as a function of logarithm of the scan rate, and variation of the Nicholson dimensionless parameter (ψ) as a function of inverse square root of the scan rate (PDF)
