# Supplementary material for: Disposable Paper-Based Biosensors: Optimizing the Electrochemical Properties of Laser-Induced Graphene
Source: ACS Appl Mater Interfaces. 2022 Jun 29;14(27):31109–20. doi: 10.1021/acsami.2c06350 (PMC9284512; doi:10.1021/acsami.2c06350)
Supplement: Supplementary file 1 — am2c06350_si_001.pdf [file am2c06350_si_001.pdf]

Supporting Information for:

Disposable Paper-based Biosensors: Optimising electrochemical properties of Laser-Induced Graphene

Gourav Bhattacharya[1*], Sam J Fishlock[1*], Shahzad Hussain[1], Sudipta Choudhury[2], Annan Xiang[3], Baljinder Kandola[3], Anurag Pritam[4], Navneet Soin[1], Susanta Sinha Roy[2], James A McLaughlin[1]

[1] School of Engineering, Ulster University, Newtownabbey, Belfast BT37 0QB, Northern Ireland, United Kingdom

[2] Department of Physics, School of Natural Sciences, Shiv Nadar University, Gautam Buddha Nagar 201314, Uttar Pradesh, India

[3] IMRI, University of Bolton, Deane Road, Bolton BL3 5AB, United Kingdom

[4] Department of Chemistry, Indian Institute of Technology Kanpur, Uttar Pradesh, 208016 India

*Corresponding authors: g.bhattacharya@ulster.ac.uk (G.B.); s.fishlock@ulster.ac.uk (S.J.F.)

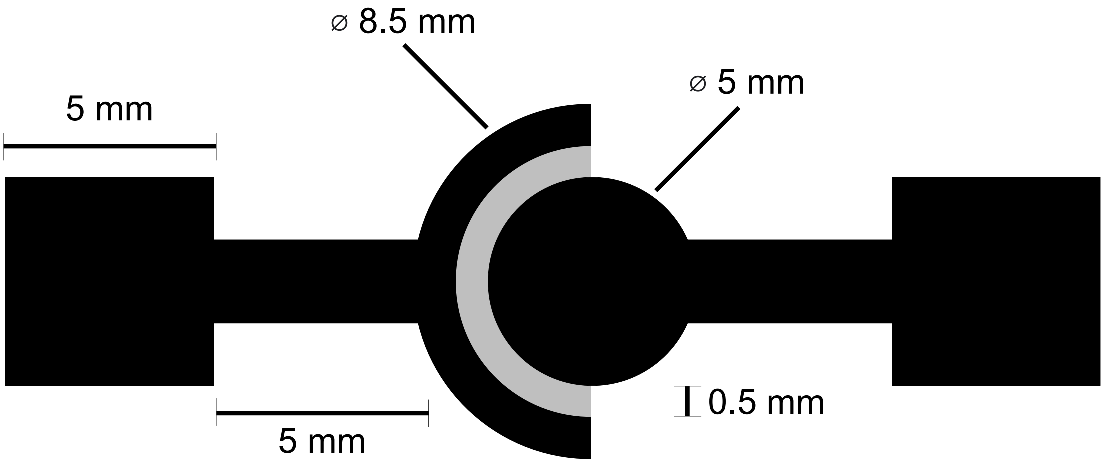

Figure S1. Geometry of the two-electrode LIG sensor. The grey section between the two electrodes is residual paper.

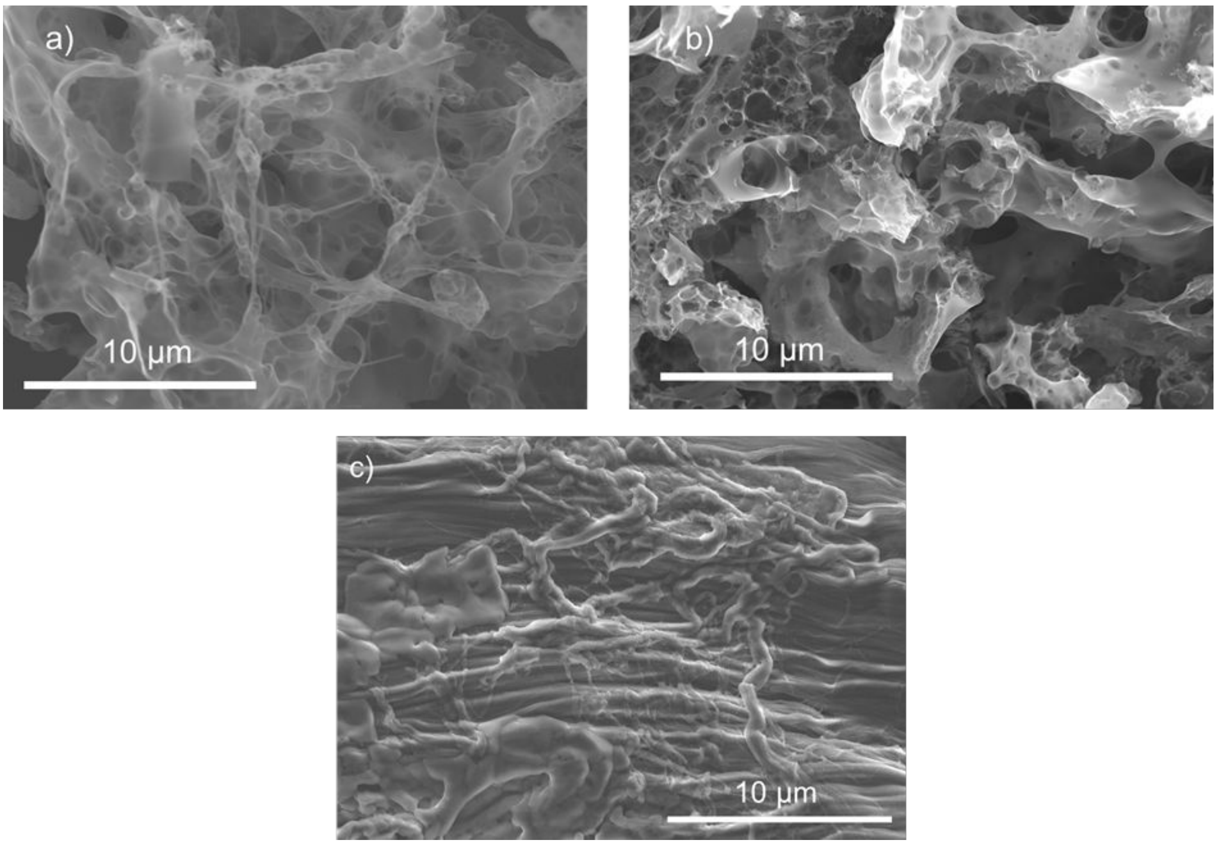

Figure S2. SEM morphological images of the LIG samples showing (a) 1F, (b) 2F (c) flame-retardant treated Whatman filter paper prior to lasing.

	Peak assignment (at.%)						
**Samples**	**C-B**	**sp^2**	**sp^3**	**C-O**	**C=O**	**O-C=O**	**π-π***
**1D**	13.64	41.16	19.36	19.03	4.29	2.51	-
**1F**	9.14	47.51	17.03	19.83	4.38	2.1	-
**1D1F**	0.9	50.15	14.13	15.29	12.55	3.93	3.06
**2F**	0.74	31.88	28.61	23.34	7.64	7.79	-

Table S1: Deconvoluted C 1s spectra for (a) 1D, (b) 1D, (c) 1D1F and (d) 2F.

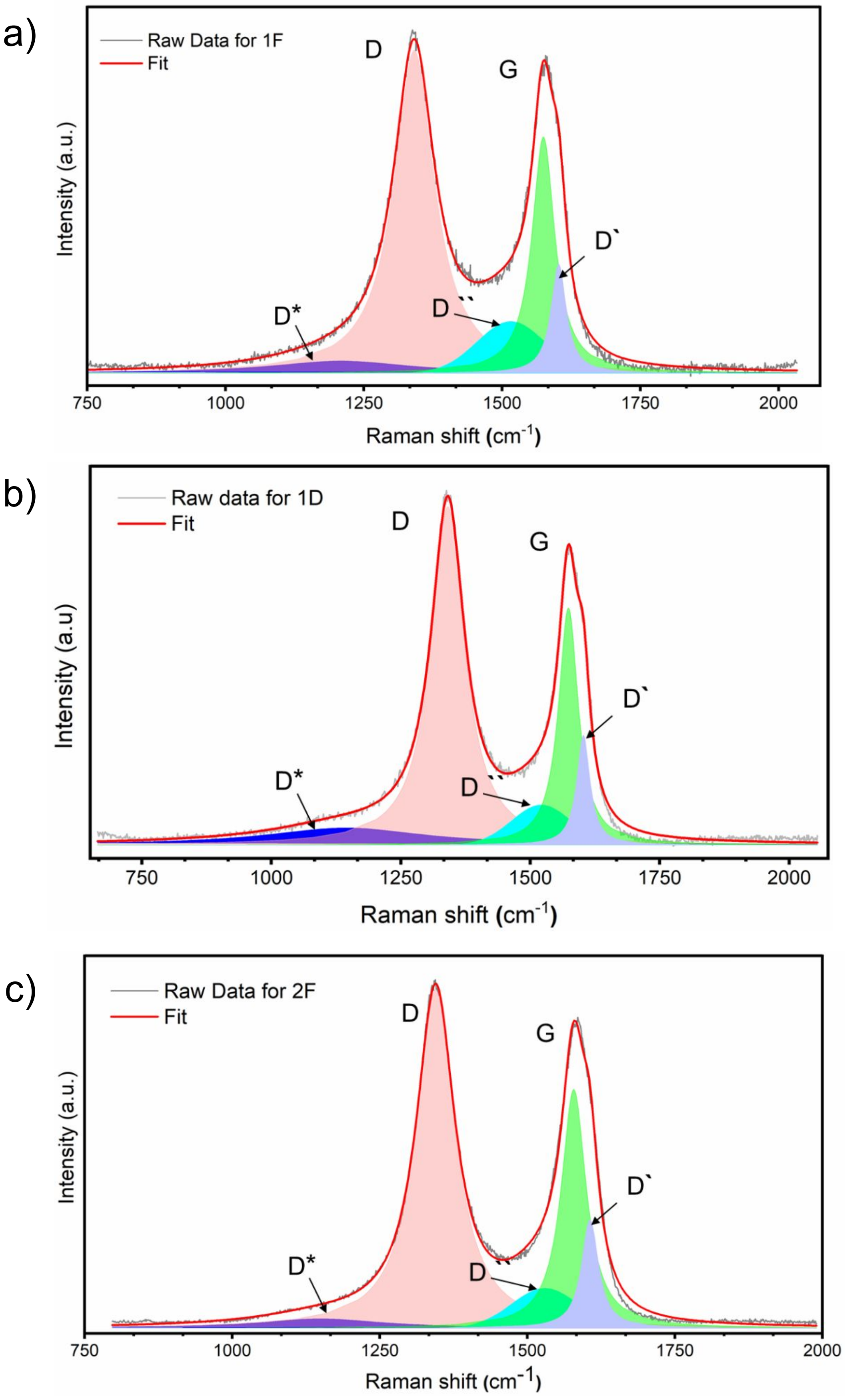

Figure S3. Deconvoluted Raman spectra using the 5 peak fitting model for (a) 1F, (b) 1D and (c) 2F samples, respectively.

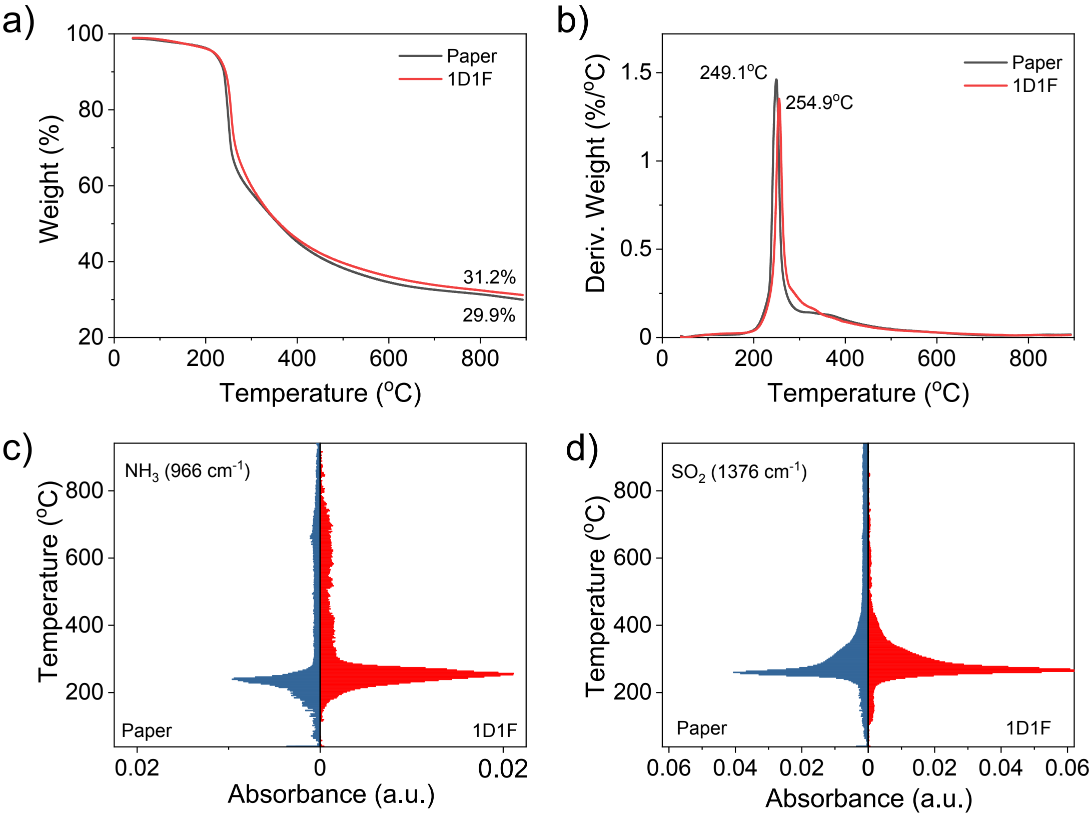

Figure S4. a-b) Pyrolysis curves of the FR-treated Whatman paper sample and the 1D1F samples. c) Releasing profile of NH_3 and d) Releasing profile of SO_2 as a function of temperature for both samples.

CH_4 was detected for both the samples within the higher temperature range of 400 – 700 ºC. Similar trend was observed in case of the release of sulphur dioxide, where the peak temperature was increased by 6 ºC for 1D1F (~260 ºC for paper and ~266 ºC for 1D1F). The higher relative absorbance of ammonia and sulphur dioxide for the 1D1F samples are in accordance with the obtained XPS results and is already discussed in the main manuscript.

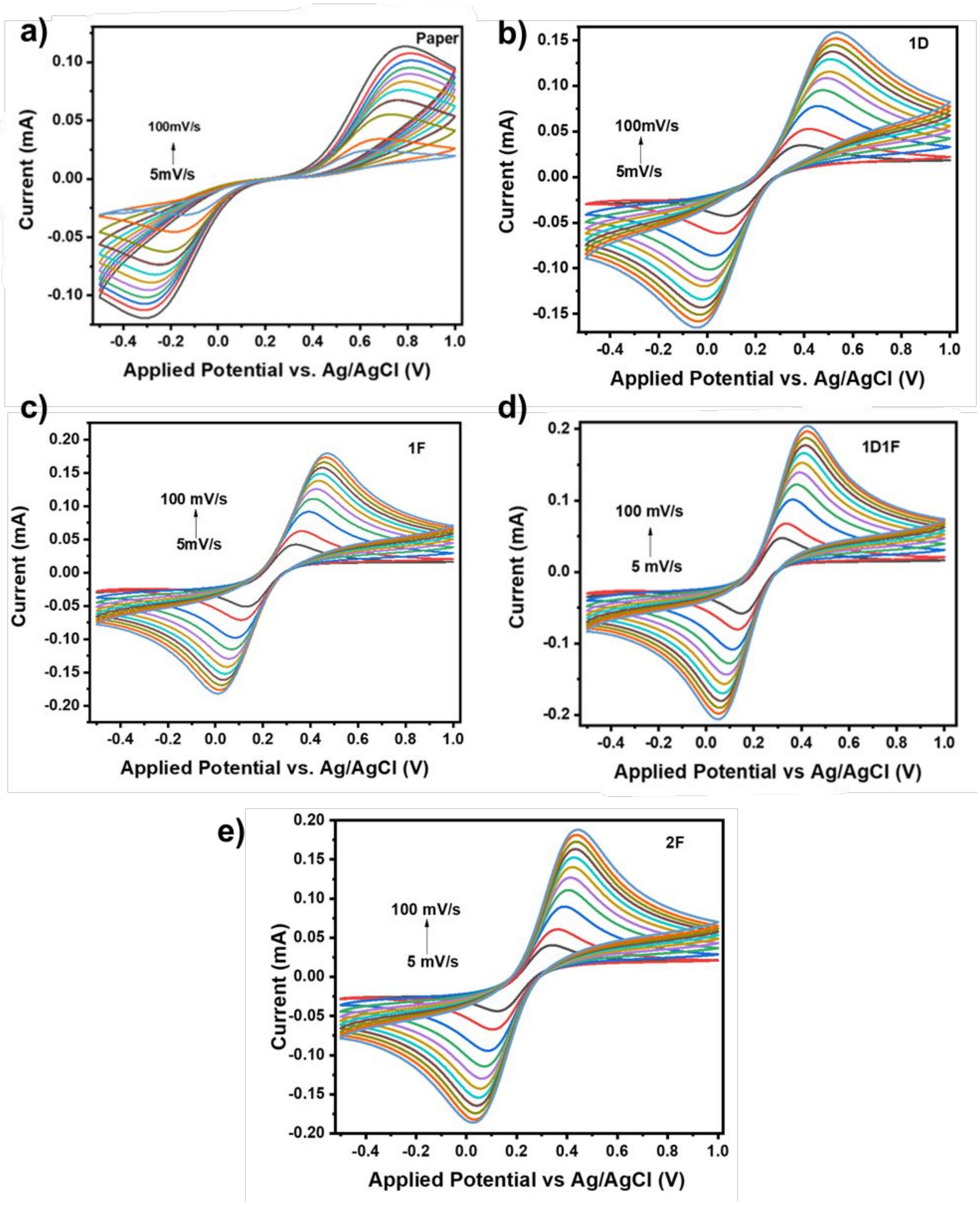

Figure S5. CV scans, from 5 to 100 mv/sec scan rate, for a) pristine paper, b) 1D, c)1F, d) 1D1F and e) 2F LIG samples, respectively.

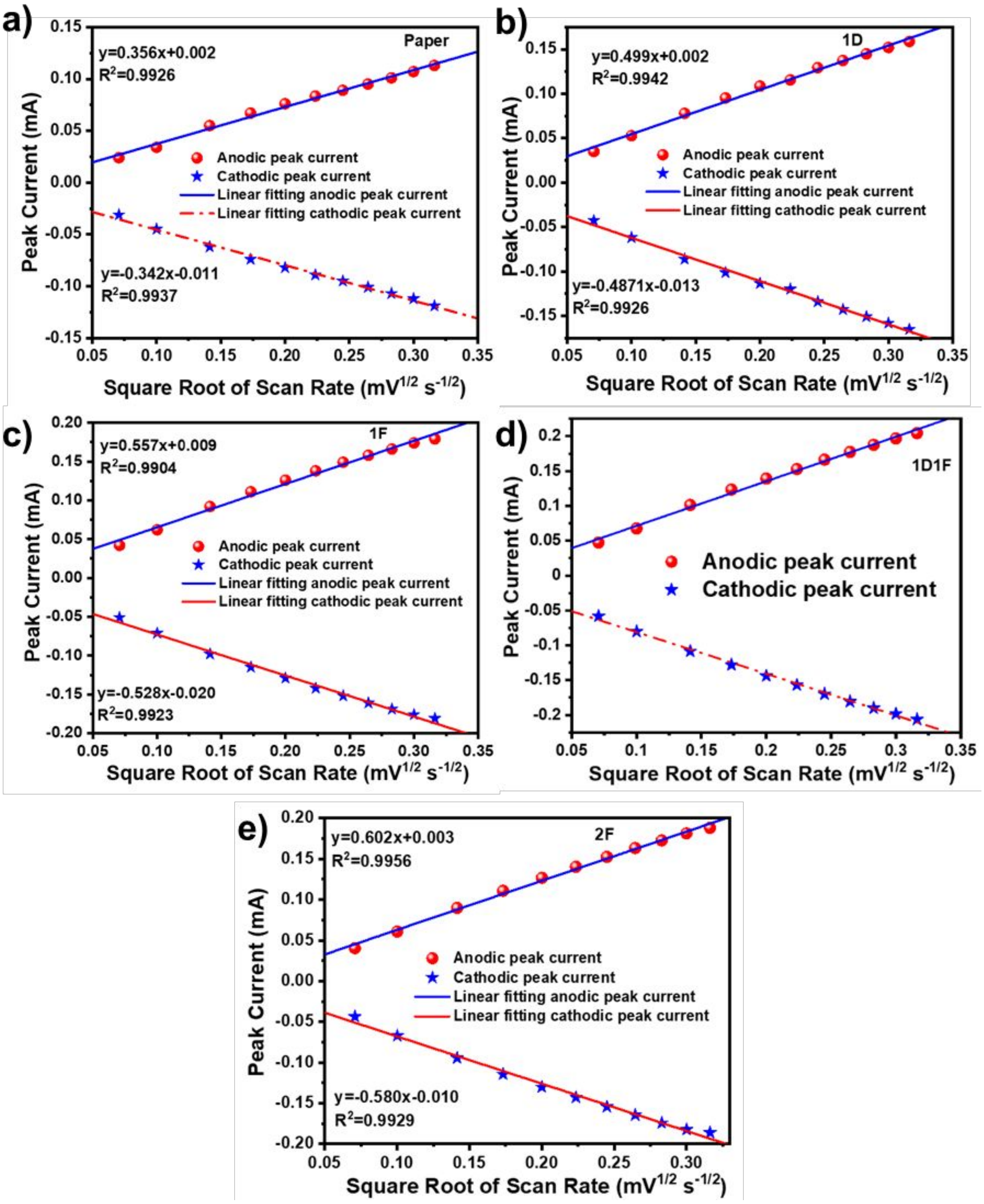

Figure S6. The anodic and cathodic peak currents, against inverse square root of scan rate, for a) pristine paper, b) 1D, c)1F, d) 1D1F, and e) 2F LIG samples, respectively.

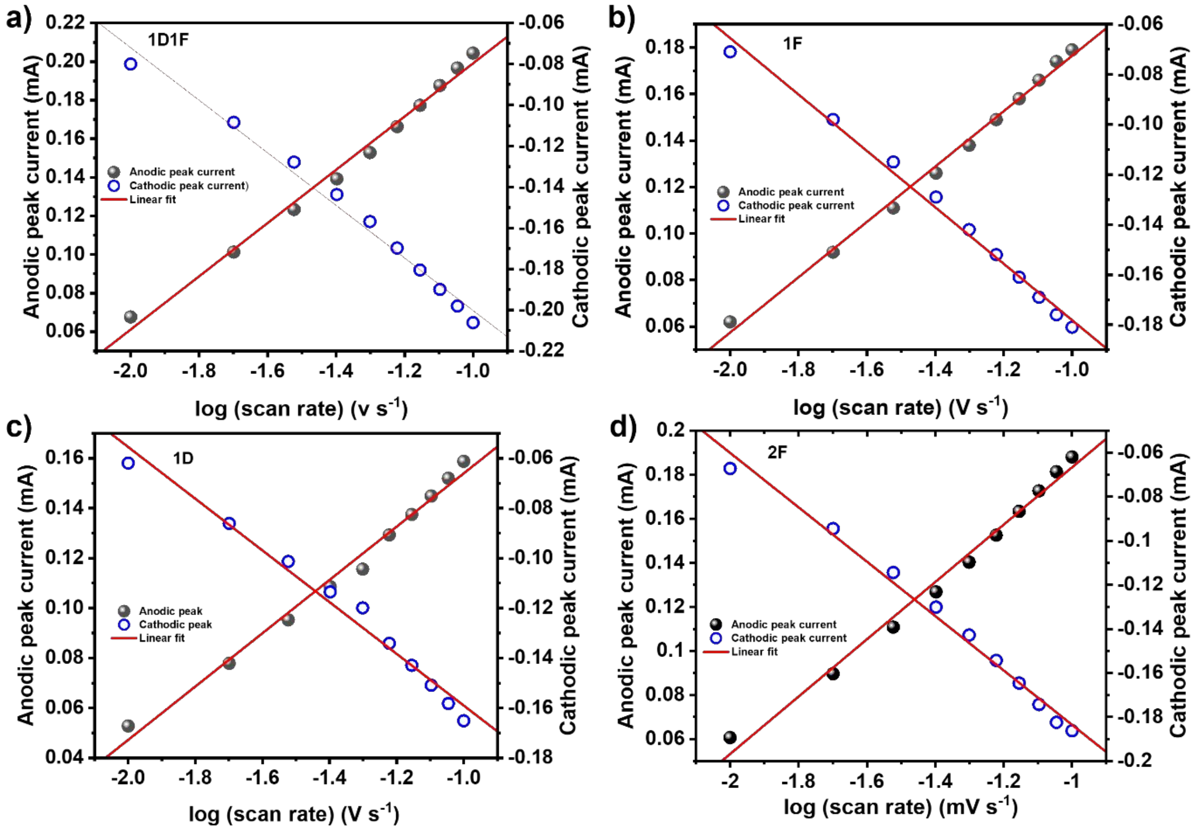

Figure S7. The anodic and cathodic peak current vs. logarithm of the scan rate plots for a) 1D, b)1F, c) 1D1F, and d) 2F LIG samples respectively.

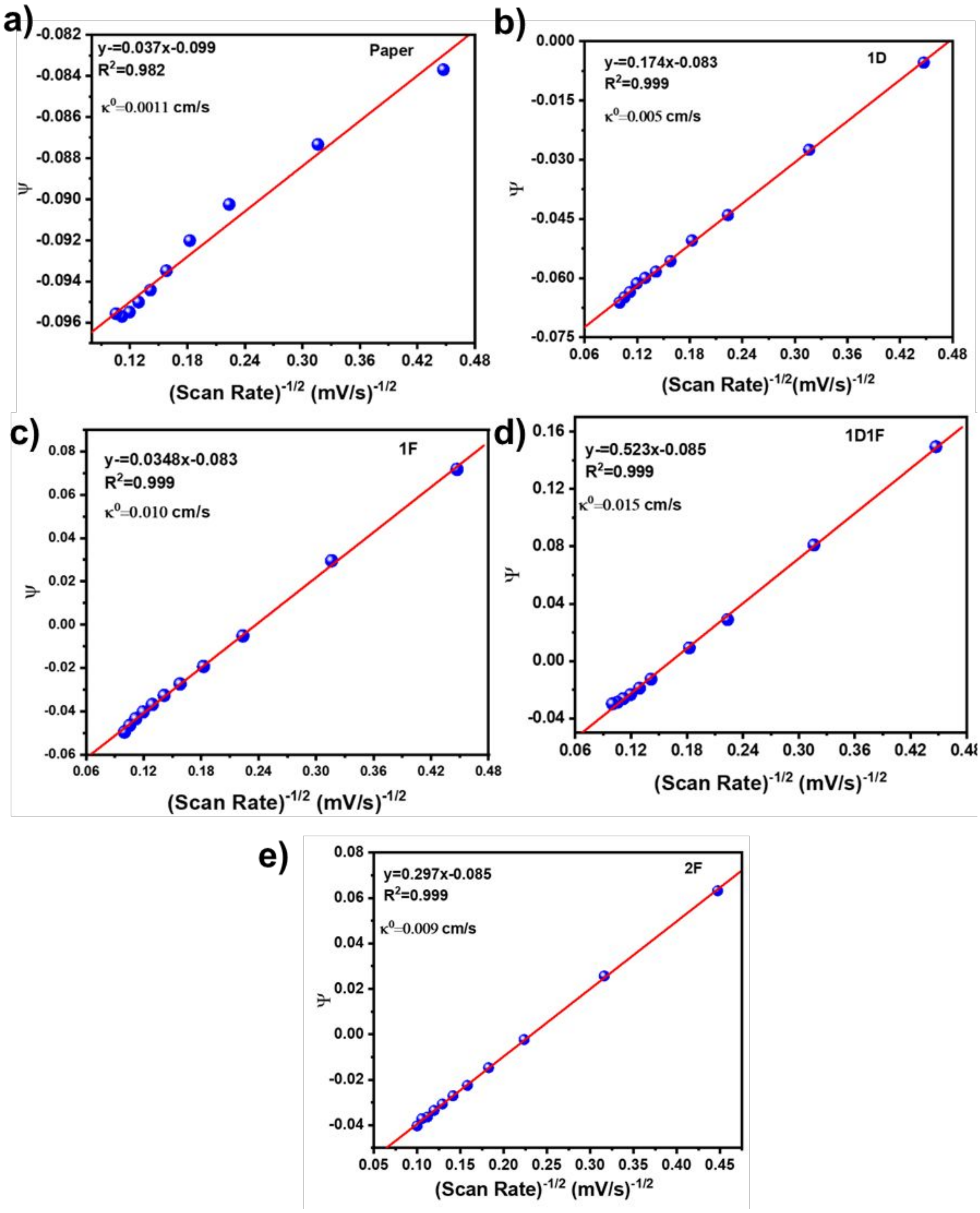

Figure S8. The parameter ψ for each sample, which is used to calculate the k_o rate constant for a) pristine, b) 1D, c)1F, d) 1D1F, and e) 2F LIG samples, respectively.
